# Death from Anæsthetics

**Published:** 1892-01

**Authors:** 


### DEATH FROM ANÆSTHETICS.

In a paper on this subject published by the *Jonrnal of the American Medical Association* of August 15, 1891, Dr. Lawrence Turnbull presents the following conclusions:

"1. During the protracted use of chloroform as an anæsthetic, the blood is changed in character, lowered in pressure, with weakening of the action of the heart and changes in its structure.

"2. Dilatation of the heart occurs under the use of chloroform at all stages, on both sides of the heart, while the heart muscle is weakened.

"3. .Cardiac failure occurred before respiration in thirteen instances out of forty-three cases of death from chloroform.

"4. The depressing influence of chloroform on the heart mechanism is not exerted through the vagus nerves, and section of both vagi does not obviate the weakening and dilating influence of chloroform on the heart.

"5. Two many trifling operations are performed under chloroform; its use should be reserved for those cases in which ether, nitrous oxide, or cocaine will not produce the anæsthesia desired.

"6. Ether death, as a rule, occurs in patients of a certain class, usually from obstructed respiration, and occasionally the heart will stop first, as in two of the fonr cases in our tables

"7. Watch both pulse and respiration, both in chloroform and ether; when the breathing becomes very rapid, danger is near.

"These changes are apt to follow the first act of respirati on Chloroform vapor should not be employed over 4 per cent."—*Brooklyn Medical Jonrnal.*

